# Targeting the RNA-Binding Protein *QKI* in Myeloid Cells Ameliorates Macrophage-Induced Renal Interstitial Fibrosis

**DOI:** 10.3390/epigenomes4010002

**Published:** 2020-02-13

**Authors:** Ruben G. de Bruin, Gillian Vogel, Jurrien Prins, Jacques M. J. G. Duijs, Roel Bijkerk, Hendrik J. P. van der Zande, Janine M. van Gils, Hetty C. de Boer, Ton J. Rabelink, Anton Jan van Zonneveld, Eric P. van der Veer, Stéphane Richard

**Affiliations:** 1 Einthoven Laboratory for Experimental Vascular Medicine, Division of Nephrology, Department of Internal Medicine, Leiden University Medical Center, Albinusdreef 2, C7-36, PO Box 9600, 2300RC Leiden, The Netherlands; rgdebruin@gmail.com (R.G.d.B.); J.Prins@lumc.nl (J.P.); J.M.G.J.Duijs@lumc.nl (J.M.J.G.D.); R.Bijkerk@lumc.nl (R.B.); H.J.P.van_der_Zande@lumc.nl (H.J.P.v.d.Z.); J.M.van_Gils@lumc.nl (J.M.v.G.); H.C.de_Boer@lumc.nl (H.C.d.B.); A.J.Rabelink@lumc.nl (T.J.R.); A.J.van_Zonneveld@lumc.nl (A.J.v.Z.); 2 Segal Cancer Center, Lady Davis Institute for Medical Research and Gerald Bronfman Department of Oncology and Departments of Biochemistry, Human Genetics and Medicine, McGill University, Montréal, QC H3T 1E2, Canada; gillianvogel@hotmail.com

**Keywords:** Quaking, RNA-binding protein, alternative splicing, macrophage, mouse, kidney diseases, post-transcriptional regulation

## Abstract

In the pathophysiologic setting of acute and chronic kidney injury, the excessive activation and recruitment of blood-borne monocytes prompts their differentiation into inflammatory macrophages, a process that leads to progressive glomerulosclerosis and interstitial fibrosis. Importantly, this differentiation of monocytes into macrophages requires the meticulous coordination of gene expression at both the transcriptional and post-transcriptional level. The transcriptomes of these cells are ultimately determined by RNA-binding proteins such as QUAKING (QKI), that define their pre-mRNA splicing and mRNA transcript patterns. Using two mouse models, namely (1) *quaking viable* mice (*qk^v^*) and (2) the conditional deletion in the myeloid cell lineage using the lysozyme 2-Cre (*QKI^FL/FL;LysM-Cre^* mice), we demonstrate that the abrogation of *QKI* expression in the myeloid cell lineage reduces macrophage infiltration following kidney injury induced by unilateral urethral obstruction (UUO). The *qk^v^* and *QKI^FL/FL;LysM-Cre^* mice both showed significant diminished interstitial collagen deposition and fibrosis in the UUO-damaged kidney, as compared to wild-type littermates. We show that macrophages isolated from *QKI^FL/FL;LysM-Cre^* mice are associated with defects in pre-mRNA splicing. Our findings demonstrate that reduced expression of the alternative splice regulator QKI in the cells of myeloid lineage attenuates renal interstitial fibrosis, suggesting that inhibition of this splice regulator may be of therapeutic value for certain kidney diseases.

## 1. Introduction

The QUAKING RNA-binding proteins belong to the family of KH-type RNA-binding proteins [1,2]. The *qkI* gene expresses three major alternatively spliced mRNAs (five, six and seven kb) encoding QKI-5, QKI-6 and QKI-7 that differ in their C-terminal 30 amino acids [3]. The QKI-5 isoform contains a unique stretch of basic amino acids at its C-terminus that functions as a nuclear localization signal [4]. QKI belongs to the heteronuclear ribonucleoprotein particle K (hnRNP K) homology (KH) domain family of RNA-binding proteins. QKI binds specific RNA sequences with high affinity termed a QKI response element (QRE) with sequence an ACUAAY (1–20) UAAY (Y; C/U) [5]. The QKI isoforms dynamically alter the post-transcriptional landscape in many cell types including oligodendrocytes, endothelial cells, (smooth) muscle cells, monocytes and macrophages by regulating RNA processing including pre-mRNA splicing [3,6,7,8].

Whole body knockout of *qkI* is embryonic lethal [9]. However, a mouse model termed the *quaking viable* mouse (*qk^v^*) contains a 1 Mb deletion in the *qkI* promoter region [10]. When recessive it leads to ubiquitous reduced levels of *qkI* mRNAs and proteins, subtly reducing QKI-5, and almost completely ablating QKI-6 and -7 expression [3,11]. Bone marrow transplanted from *qk^v^* mice into LDLR^-/-^ mice fed a high-fat diet had reduced infiltrating macrophages in their atherosclerotic lesions [12]. Our previous study reported QKI proteins as key players in the post-transcriptional process defining the identity of monocyte and macrophages and their proper function including cell adhesion, migration and phagocytosis [12]. Interestingly, a patient was identified that harbors a deletion within *qkI* causing QKI haploinsufficiency [13]. Monocytes from this patient had reduced adhesion and migration potential, demonstrating a role for QKI in monocyte/macrophage function [12]. The altered expression of the QKI RNA-binding protein has also been shown to be associated with cancer including angiocentric gliomas, as well as schizophrenia, atherosclerosis and vascular stenosis [11,14,15,16]. Whether QKI plays a role in kidney disease has *hitherto* not been investigated.

In the pathophysiologic setting of acute and chronic kidney injury, the recruitment of circulating monocytes has been extensively described to play a central role in driving glomerulosclerosis and interstitial fibrosis [17,18,19]. The chronic and progressive sclerosis of the kidney, commonly known as chronic kidney disease (CKD) can lead to end-stage renal disease caused by e.g., diabetes, hypertension, ischemia reperfusion injury following kidney transplantation and acute or chronic allograft rejection [20,21]. A fibrotic process is directly associated with decreased renal function, necessitating renal-replacement therapies such as dialysis or organ transplantation [22,23]. Therefore, gaining a better understanding of macrophage-induced kidney injury, and their role in the initiation, maintenance and progression of CKD to end-stage renal disease could provide new insights for generating therapeutic strategies to prevent loss of renal function.

Circulating monocytes serve as potent mediators of inflammation and organ damage by their homing, extravasation and differentiation into tissue macrophages at sites of injury [24,25]. Depending on the local milieu, monocytes differentiate into macrophages that are characterized by distinct capacities to generate cytokines, phagocytose dead cells and debris, and instruct adaptive immune responses by presenting antigens. A subset of these circulating monocytes, namely non-classical monocytes, has been implicated in directing processes that are geared toward resolving detrimental organ dysfunction by promoting angiogenesis, tissue remodeling and scar-tissue formation [26]. This well-orchestrated reparative response ideally restores tissue architecture and organ function.

Previously, we demonstrated that the RNA-binding protein QKI serves an essential role in orchestrating the transcriptome of monocytes and macrophages by governing pre-mRNA splicing and gene expression [12]. Given that monocyte recruitment and infiltration into the damaged kidney leading to local inflammation and phagocytosis by macrophages is associated with kidney injury and fibrotic responses, we sought to determine if the specific abrogation of QKI in monocytes could attenuate tissue injury and interstitial fibrosis. Herein, we report that QKI-deficiency in monocytes *in vivo*, using both *quaking viable* mice and a conditional allele of *qkI* in mice, led to a reduction in injury, inflammation and renal interstitial fibrosis upon unilateral urethral obstruction. We postulate that the modulation of the cellular transcriptome by interfering with QKI RNA-binding proteins represents a new and potent means of altering monocyte/macrophage responses and could aid in the prevention of kidney diseases.

## 2. Results

### 2.1. Abundant Expression of the QKI RNA-Binding Protein in the Kidney

**Figure 1:**
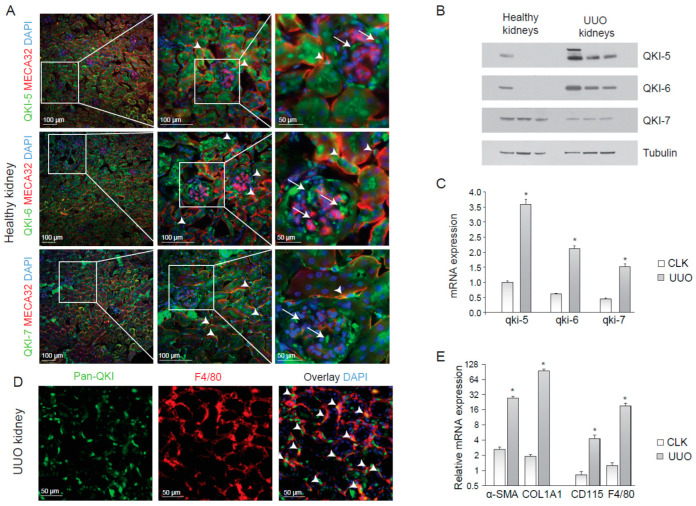
QUAKING (QKI) is expressed in infiltrating macrophages upon unilateral urethral obstruction. (**A**) Immunostaining of mouse kidney cryosections for QKI-5 (green, upper panels), QKI-6 (green, middle panels) and QKI-7 (green, lower panels). Endothelial cells are stained using the MECA-32 antibody in red. Nuclei are stained blue using DAPI. (**B**) Western blot analysis using whole kidney lysates prepared from (*n* = 3) healthy contralateral kidneys (healthy kidneys) and unilateral urethral obstruction (UUO) kidneys were performed using anti-QKI-5, -QKI-6, -QKI-7 and -tubulin antibodies. (**C**) Whole kidney lysates of either contralateral kidneys (CLK) or UUO kidneys were assessed for mRNA levels of fibrosis markers (α-SMA, COL1A1) and macrophage markers (CD115, F4/80). (**D**) Whole kidney lysates were assessed for QKI mRNA levels using qRT-PCR in healthy contralateral kidneys (CLK) as compared to fibrotic kidneys 10 days after UUO. (**E**) Immunostaining for Pan-QKI (green) and F4/80 (macrophage marker in red) on cryosections of fibrotic kidneys 10 days after UUO. * *p* ≤ 0.05 by Students’ *t*-test, error bars represent the standard error of the mean (SEM).

To determine the expression of the QKI isoforms within the kidney, we performed immunohistochemistry on cryosections of healthy mouse kidneys using antibodies that specifically detect the individual QKI-5, QKI-6 and QKI-7 isoforms, together with the endothelial cell marker MECA-32. As illustrated in Figure 1A, all three QKI isoforms were expressed in tubular epithelial cells, although some tubuli appeared void of QKI-7. Endothelial cells (arrowheads) and a few striking intraglomerular cells (arrows) that were MECA-32 negative, did express QKI. The latter are too few in number to represent either mesangial cells or podocytes. QKI-5 and QKI-6 display nuclear enrichment within renal cells, but were also abundantly detected in the cytoplasm of the tubular epithelium, consistent with their known cellular localizations [15]. In contrast, QKI-7 was primarily localized in the peri-nuclear compartment and the cytoplasm (Figure 1A; arrows lower panels). Given that QKI is regarded as a global regulator of pre-mRNA splicing [11] and thus affecting the total cellular transcriptome, the expression of QKI in individual cell types within the kidney could impact their transcriptome and thereby cellular function in a disease setting.

### 2.2. Kidney Injury Induces an Influx of QKI-Expressing Macrophages

Macrophage infiltration during kidney disease is known to contribute to inflammation and fibrosis [24,25]. To examine whether QKI-expressing macrophages were involved in the inflammatory response during renal injury, we performed unilateral urethral obstruction (UUO) in wild-type C57BL6 mice, a kidney injury model that is associated with tubular cell injury, inflammation and elaborate interstitial fibrosis [27]. After 10 days, we harvested protein and RNA and tissue from the damaged (UUO) kidney and contralateral kidney (CLK). The antibodies were specific for each QKI isoform, as determined by immunoblotting (Figure 1B). Interestingly, the expression of both isoform, QKI-5 and QKI-6, which have access to the nucleus, were increased during UUO (Figure 1B). Furthermore, the mRNA expression levels of all three *qkI* isoforms significantly increased in fibrotic kidneys, as compared to healthy contralateral kidneys (Figure 1C, gray bars). This increase is likely the result of an influx of inflammatory cells such as bone marrow-derived monocytes and macrophages in the UUO-damaged kidneys. Indeed, immunohistochemical analysis revealed expression of the distinct QKI isoforms in infiltrating macrophages, as evidenced by pan anti-QKI antibody staining (detects all three QKI isoforms) and F4/80 co-localization in UUO-damaged kidneys (Figure 1E, arrowheads). As expected, we detected increased production of the extracellular matrix protein collagen 1A1 (COL1A1), as well as enhanced intracellular expression of the fibroblast-marker smooth-muscle alpha actin (α-SMA) in UUO kidneys (Figure 1D), confirming the induction of a vast fibrotic response. An increase in mRNA expression of F4/80 and CD115, markers of bone marrow-derived monocytes and macrophages, confirmed an influx of inflammatory cells during injury (Figure 1E, gray bars).

### 2.3. QKI Viable Mice (qk^v^) Show Decreased Interstitial Fibrosis upon UUO

**Figure 2:**
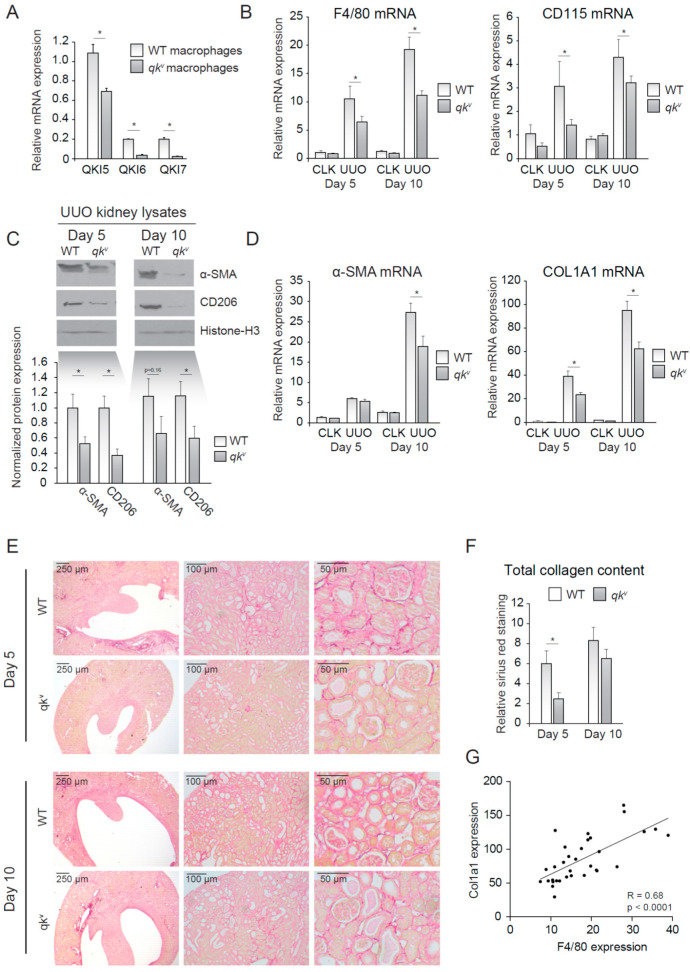
Quaking viable mice (*qk^v^*) display decreased macrophage influx and decreased interstitial fibrosis upon unilateral urethral obstruction (UUO). (**A**) mRNA expression of QKI mRNA levels in cultured macrophages of either *qk^v^* or wild-type littermate controls. (**B**) mRNA levels of macrophage markers (F4/80, CD115) in whole kidney lysates derived from either *qk^v^* mice (gray bars) or wild-type littermate controls on day 5 and 10 after UUO. (**C**) Western blot analysis of whole UUO kidney lysates for α-SMA, CD206 and histone H3. Quantitation using ImageJ software is provided below. (**D**) mRNA levels of fibrosis markers (α-SMA and COL1A1) in whole kidney lysates derived from either *qk^v^* mice (gray bars) or wild-type littermate controls (open bars) on day 5 and 10 after UUO. (**E**) Representative photomicrographs of Sirius Red staining for collagen on 4 μm sections of 5- or 10-day UUO kidneys from either *qk^v^* or wild-type littermate controls. (**F**) Quantitation of Sirius red staining is provided. (**G**) Pearson R correlation is plotted for COL1A1 and F4/80 expression. * *p* ≤ 0.05 by Students’ *t*-test, error bars represent the SEM.

We next investigated whether reduced QKI expression in monocytes/macrophages offered protection against kidney injury induced by UUO. In vitro M-CSF stimulated bone marrow-derived mouse macrophages isolated from wild-type mice expressed abundant levels of all three of the *qkI* mRNAs with the nuclear isoform *qkI-5* being the most elevated (Figure 2A, open bars). This expression pattern was reduced in *qk^v^* mice (Figure 2A, closed bars), a mouse model of reduced QKI expression [10]. This partial maintenance of QKI-5 expression is expected for *qk^v^* mice, as the *qkI* promoter/enhancer deletion has been shown to affect all isoforms within a particular prominent effect on the expression of the cytoplasmic QKI-6 and QKI-7 isoforms [15]. We next induced kidney damage using UUO and compared the damaged kidney with its healthy counterpart (CLK). Importantly, fibrotic kidneys harvested from wild-type littermate controls displayed abundant expression of the macrophage-specific markers F4/80 and CD115 at day 5 and 10 following UUO, consistent with macrophage infiltration (Figure 2B). In contrast, the *qk^v^* mouse fibrotic kidneys were characterized by significantly lower mRNA levels of macrophage-specific markers F4/80 and CD115 (Figure 2B). These results suggested that reduced QKI expression in monocytes diminished their kidney influx and subsequent differentiation into macrophages. The reduction in macrophage content was validated by Western blot analysis of whole kidney lysates for the macrophage marker CD206, in which decreased expression was observed in *qk^v^* UUO-damaged kidneys (Figure 2C, quantitation of *n* = 7 mice is presented below). Given the attributing role of infiltrating macrophages in inducing renal fibrosis, we assessed whether mRNA levels of the fibrosis markers α-SMA and COL1A1 were similarly reduced in *qk^v^* UUO kidneys. Indeed, these markers were substantially less abundant in *qk^v^* UUO kidneys on the mRNA (Figure 2D) and protein level, as assessed by Western blot (Figure 2C, upper blot, quantitation of *n* = 7 mice is presented below). To further substantiate these anti-fibrotic effects observed in *qk^v^* UUO kidneys, we performed Sirius red staining of paraffin-embedded kidney sections to visualize and quantitate all interstitially deposited collagens. These studies clearly showed decreased interstitial fibrosis, as evidenced by less collagen staining in *qk^v^* UUO kidneys as compared to wild-type littermate control UUO kidneys (representative photomicrographs are presented in Figure 2E and colorimetric quantitation in Figure 2F). Moreover, a strong correlation between F4/80 and COL1A1 mRNA expression existed (Pearson *R* = 0.68, *p* < 0.0001, Figure 2G). Taken together, these data suggest that reduction of QKI expression leads to reduced macrophage influx and subsequently reduced interstitial fibrosis of the kidney.

### 2.4. QKI^FL/FL;LysM-Cre^ Mice Show Decreased Interstitial Fibrosis upon UUO Kidney Damage

**Figure 3:**
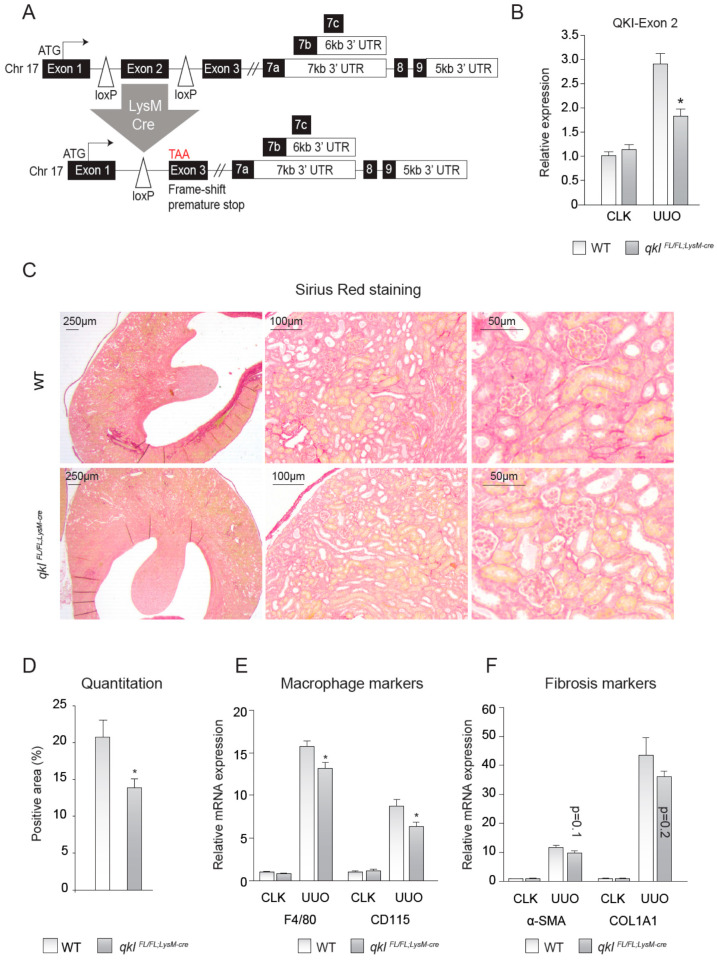
Monocyte-specific knockout of QKI ameliorates interstitial fibrosis upon unilateral urethral obstruction (UUO). (**A**) Schematic representing the genomic architecture of the conditional QKI knockout mouse (not to scale). (**B**) Relative expression level of the mRNA levels of the floxed exon 2 of the QKI mRNA in 10-day CLK and UUO kidneys normalized to the mRNA of β-actin is shown. (**C**) Sirius red staining for collagen in 4 μm sections of 10-day UUO kidneys derived from *qkI*^FL/FL;LysM-cre^ or wild-type littermate controls. (**D**) Colorimetric quantitation of Sirius Red staining using ImageJ software. (**E**) mRNA levels of macrophage markers (CD115, F4/80) in whole kidney lysates derived from either *qkI*^FL/FL;LysM-cre^ mice (gray bars) or wild-type littermate controls (open bars) on day 10 after UUO. (**F**) mRNA levels of fibrosis markers (α-SMA, COL1A1) in whole kidney lysates derived from either *qkI*^FL/FL;LysM-cre^ mice (gray bars) or wild-type littermate controls (open bars) on day 10 after UUO. * *p* ≤ 0.05 by Students’ *t*-test, error bars represent SEM.

To test whether the observed effects were cell autonomous to monocytes and macrophages and not the result of a reduction of QKI in e.g., interstitial fibroblasts or renal epithelial cells, we generated *QKI^FL/FL;LysM-Cre^* mice, yielding a monocyte/macrophage-specific reduction of QKI (Figure 3A). These genetically modified *QKI^FL/FL;LysM-Cre^* mice constitutively express a Cre-recombinase driven by the LysM promoter (also described as Lyz2), yielding monocyte/macrophage-specific Cre-recombinase expression [28]. The presence of loxP sites flanking the genomic DNA sequence encoding exon 2 of the *qkI* gene induces an exonic frame-shift that generates a premature stop codon in exon 3 [3], leading to the monocyte-specific abrogation of all QKI protein isoforms that are generated by extensive alternative splicing of their 3′ end (as illustrated in Figure 3A). Although the LysM-driven LoxP-recombination event does not terminate transcription of the *qkI* locus in the non-myeloid cells of the kidney, mRNA levels of the floxed exon 2 were significantly reduced in whole kidney lysates of *QKI^FL/FL;LysM-Cre^* UUO kidneys (Figure 3B), suggesting that the residual exon 2 expression is likely contributed by non-macrophage infiltrating cells. Moreover, this demonstrates that the recombination event is specifically induced in infiltrating cells in UUO kidneys and not in contralateral kidneys that are void of infiltrating cells. We subsequently assessed whether these mice are protected from UUO-induced kidney fibrosis, similar to the *qk^v^* mice. Indeed, *QKI^FL/FL;LysM-Cre^* mice also showed reduced interstitial fibrosis as evidenced by decreased Sirius red staining (representative photomicrographs are shown in Figure 3C and quantitation is provided in Figure 3D). Furthermore, mRNA levels of the macrophage markers F4/80 and CD115 were significantly reduced in *QKI^FL/FL;LysM-Cre^* fibrotic kidneys (Figure 3E), whereas the fibrosis genes α-SMA and COL1A1 only showed a trend toward reduced expression levels in *QKI^FL/FL;LysM-Cre^* UUO kidneys (Figure 3F). These data further support our hypothesis that QKI expression levels in monocytes and macrophages contribute to the formation of interstitial fibrosis upon UUO kidney damage.

### 2.5. QKI Mediates Alternative Splicing in Mouse Macrophages

**Figure 4:**
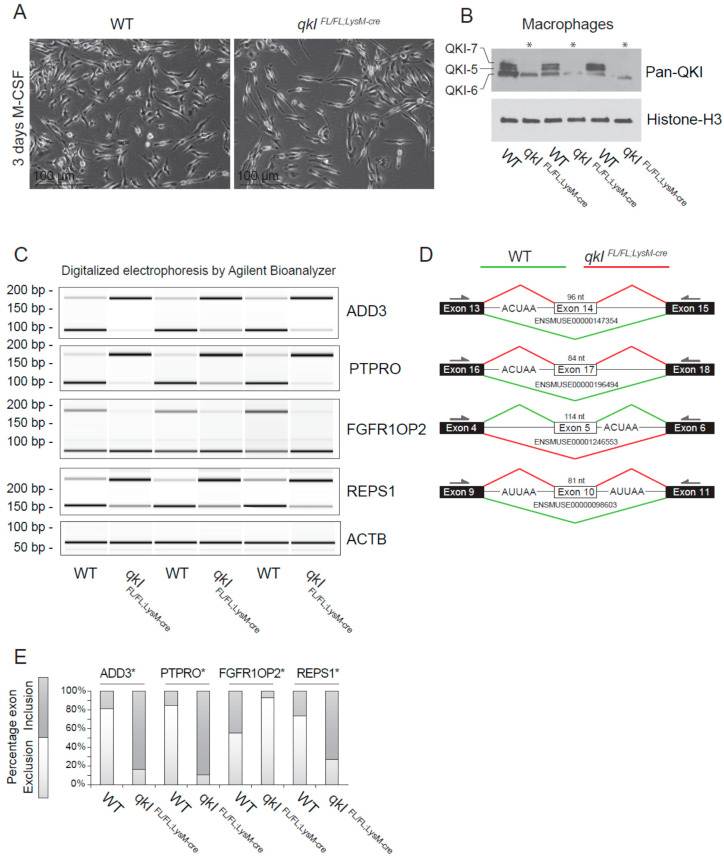
QKI mediates alternative splicing in macrophages. (**A**) Photomicrographs of cultured bone marrow-derived macrophages 3 days after M-CSF stimulation. (**B**) Western blot analysis of cultured mouse macrophages from *qkI*^FL/FL;LysM-cre^ or wild-type littermate controls using a pan-QKI (N-terminal) antibody and histone H3 loading control. (**C**) PCR analysis of cassette exon alternative splicing in 5 days M-CSF stimulated cultured macrophages derived from either wild-type or *qkI*^FL/FL;LysM-cre^ mouse bone marrow. Gel electrophoresis of PCR products generated using primers in flanking exons are shown as digitalized electrophoresis by Agilent Bioanalyzer, illustrating the abundance of alternative mRNA isoforms expressed. (**D**) In silico analysis of the particular exon assessed illustrates the predicted alternative splicing event based on QRE position (defined as an ACUAA/AUUAA motif) and denotes the predominant splicing event in wild-type (green line) or *qkI*^FL/FL;LysM-cre^ (red line) macrophages. * *p* ≤ 0.05 by Students’ *t*-test. (**E**) Densitometric quantification of bands in panel (C) using ImageJ software and statistical testing is provided for the assessed splicing events.

Previously, we provided evidence that QKI governs alternative splicing of pre-mRNA transcripts in human macrophages that affect their adhesion and ability to migrate [12]. To confirm whether QKI regulates alternative splicing in mouse macrophages, we isolated total RNA from wild-type and *QKI^FL/FL;LysM-Cre^* cultured bone marrow-derived macrophages (Figure 4A) and performed RT-PCR. We initially examined the ability of the *QKI^FL/FL;LysM-Cre^* to fully remove the QKI-5, QKI-6 and QKI-7 isoforms. Indeed deletion of *qkI* exon 2 deletes all three isoforms [3,29], as detected using a pan-QKI antibody. This antibody was generated against the common region of the QKI isoforms and also recognizes, at a low level, some other unknown RNA-binding proteins [30] remaining in the *QKI^FL/FL;LysM-Cre^* macrophages. We chose genes for the cytoskeletal protein γ-adducin (ADD3), protein tyrosine phosphatase receptor type O (PTPRO), fibroblast growth factor receptor 1 oncogene partner 2 (FGFR1OP2) and RalBP1-associated Eps domain-containing protein 1 (REPS1), as each gene is known to harbor a QRE neighboring the splicing event [12]. Indeed QKI-deficient macrophages displayed exon inclusion for ADD3, PTPRO and REPS1, and exon exclusion for FGFR1OP2 (Figure 4C,D, quantitation in Figure 4E). These findings show that QKI-deficiency in mouse macrophages influences the transcriptome by regulating alternative splicing.

## 3. Discussion

In the present manuscript, we show that the abrogation of *QKI* expression in the myeloid cell lineage using the *quaking viable* mice (*qk^v^*) and lysozyme 2-Cre (*QKI^FL/FL;LysM-Cre^* mice) diminished macrophage infiltration into the UUO-damaged kidney. The *qk^v^* and *QKI^FL/FL;LysM-Cre^* mice showed a significant diminished interstitial collagen deposition and fibrosis, as compared to wild-type littermates. Furthermore, we show that macrophages isolated from *QKI^FL/FL;LysM-Cre^* mice are associated with defects in pre-mRNA splicing that affect the macrophage transcriptome. Previously, we have shown that perturbation of splicing in monocytes impacts their capacity to become bona fide macrophages [12]. Here, we show that reduced expression of the alternative splice regulator QKI in cells of myeloid lineage limits renal interstitial fibrosis. Thus, QKI RNA-binding proteins may be of therapeutic value for certain kidney diseases.

At present, abrogating the expression or activity of RNA-binding proteins does not yet belong to the list of therapeutic modalities utilized for limiting disease progression in general. This could potentially be due to their upstream hierarchical position in guiding cellular transcriptomes in health and disease. Nonetheless, our data suggest that targeting QKI in the monocyte could be an effective means of limiting their adhesion, extravasation and differentiation at sites of kidney injury. To achieve this goal, several technologies could be implemented, namely: (1) naked or lipid-encapsulated siRNAs to target individual RNA-binding proteins (reviewed in [31]); (2) pRNAs and RNA aptamer-based approaches [32,33,34]; and (3) RNA-based antisense oligonucleotides. Importantly, QKI does not represent the sole RNA-binding protein that is active in monocytes and macrophages, as a diverse repertoire of such proteins are active and meticulously determining the transcriptome, including AU-rich element-binding proteins (ARE-BPs) that in particular interact with cytokine mRNAs [35,36] and Roquins, which also drive mRNA decay processes [37]. These RNA-binding proteins, in concert with RNA-binding proteins such as QKI [11], CELF1 [38] and MBNL1 [39], are responsible for triggering both changes in monocyte/macrophage function and their mediation of pathophysiological processes.

ADD3, a structural cytoskeletal protein that influences the cortical actin layer [40], could very well influence the homing of monocytes to the injured kidney. Moreover, PTPRO, a tyrosine phosphatase, has been shown to localize to the cellular “integrin adhesome” [41], which is essential in monocyte adhesion and migration [42]. PTPRO has furthermore been implicated in ischemia reperfusion injury of the liver by activating Nuclear factor-κB (NF-κB) [43] and thereby the classical or non-classical activation of monocytes/macrophages [44,45]. Interestingly, FGFR1OP2 has been extensively investigated in the context of hematopoietic malignancies including acute myeloid leukemia and myelomonocytic leukemia where it is involved in monocyte precursor differentiation in the bone marrow [46,47,48]. The conclusion that the described alternative splicing events and their resulting protein isoforms contribute to proper physiological monocyte/macrophage function is likely, but remains to be fully elucidated.

It is tempting to speculate that a reduction of QKI expression levels not only affects the phenotype of monocytes and infiltrated macrophages (for review see [11]), but could also skew these cells toward a tissue-regenerative and fibrosis-ameliorating function. Although some controversy still exists about the exact role of monocytes and macrophages in organ fibrosis, it has been suggested that macrophages can also contribute to the resolution of fibrosis by affecting myofibroblast function and enhancing matrix metalloproteinase (MMP) and collagenase expression, while concomitantly decreasing the expression of tissue inhibitors of MMPs (TIMPs) [49]. While numerous studies have provided evidence that organ fibrosis can be reversed [50,51,52,53,54,55,56], a limited number of studies have actually shown regression or resolution of fibrotic lesions in the renal compartment [57,58,59]. Recently, Sugimoto and colleagues provided evidence of effective renal fibrosis resolution following the administration of an activator of Activin-like kinase 3 (ALK3/BMPR1A), namely THR-123 [60]. Similar to our observation that a reduction of QKI in a monocyte-specific fashion reduces monocyte infiltration, inflammation and kidney fibrosis, with the THR-123 treatment regimen employed, lead to a decrease in monocyte influx and a reversal of kidney fibrosis *in vivo*. Similarly, we showed previously that modifying the transcriptome of bone marrow cells by hematopoietic overexpression of microRNA-126, exerts anti-inflammatory and anti-fibrotic effects in vivo [61]. We, therefore, propose that monocytes and macrophages are the ideal cell type of which the cellular phenotype can be tailored to ameliorate or even direct reversal of kidney fibrosis.

In conclusion, we have identified that the RNA-binding protein QKI influences the phenotype of infiltrating macrophages and their capacity to drive kidney injury. Thus, diminishing expression or reducing QKI activity in monocytes and macrophages could serve as an effective means of limiting renal fibrosis in the damaged kidney.

## 4. Material and Methods

### 4.1. Cell Culture

Mouse macrophages were derived from whole bone marrow samples of mouse femurs. Cells were plated in 6-well plates at a density of 2 × 10^6^ cells per well in RPMI medium containing 100 U/mL penicillin, 100 U/mL streptomycin, 300 μg/mL glutamine (Thermo Fisher Scientific, Waltham, MA, USA) and 10% fetal calf serum (Cambrex, East Rutherford, NJ, USA). Additionally, 10 ng/mL recombinant mouse M-CSF (Peprotech, Rocky Hill, CT, USA) was added to differentiate the cells into macrophages in 7 days. Medium was changed once after 3 days. Monocytes were isolated using MACS-technology using the CD115 positive selection kit for mouse monocytes (Miltenyi, Bergisch Gladbach, Germany).

### 4.2. Antibodies

The commercial antibodies purchased in this study are depicted in Table 1.

### 4.3. Western Blot Analysis

A quarter of a kidney was homogenized using a IKA^®^ tissue homogenizer in 500 μL RIPA buffer (Sigma Aldrich, St. Louis, MO, USA) containing Complete^®^ protease inhibitors (Roche, Basel, Switerland). Subsequently, a Pierce BCA protein assay (Thermo Fisher Scientific, Waltham, MA, USA) was performed to assess the absolute quantity of protein in each sample. Next 10–40 μg of total protein lysate was loaded per lane on precast Any-kD acrylamide gels (Bio-Rad, Hercules, CA, USA) for gel electrophoresis. Using the Trans-Blot^®^ Turbo™ Transfer system (Bio-Rad), the protein was transferred to Nitrocellulose (Bio-Rad) membranes for further analysis. Membranes were blocked and antibodies incubated o/n at 4 °C in 5% skim milk powder (Nutricia, Zoetermeer, Netherlands) in PBS. The appropriate HRP-labeled secondary antibodies (Dako, Glostrup, Denmark) were incubated for 1 h at room temperature, followed by extensive washing with PBS. Band-visualization was achieved using SuperSignal West-Dura^®^ Extended Duration Substrate (Thermo Fisher Scientific, Waltham, MA, USA) for HRP. Next, exposure on UltraCruz^®^ Autoradiography Film or KODAK-XAR film and development by a Konica developer followed. Finally, band intensities were quantified using ImageJ 1.x [62].

### 4.4. RNA Isolation, cDNA Synthesis and qRT-PCR Analysis

A quarter of a kidney was lysed in Trizol reagent (Invitrogen, Carlsbad, CA, USA) using an electrical tissue homogenizer (IKA^®^). Subsequently, chloroform and ethanol were used to phase-separate the RNA and subsequently RNeasy mini-columns (Qiagen, Hilden, Germany) were used to further purify the RNA according to manufacturers’ guidelines. The on-column DNase step was also performed to eliminate any contaminating genomic DNA. cDNA synthesis was performed using 1 μg RNA, random primers, FS-buffer, DNTPs, M-MLV-RT in the presence of RNAsin according to the manufacturers’ guidelines (all Promega products, Madison, WI, USA). RT-qPCR was performed using SYBR-select (Invitrogen) and PCR was run on a Bio-Rad CFX-384. Relative mRNA expression was calculated using the ΔΔ*C*_T_ method, and was normalized to GAPDH mRNA levels. PCR products generated by the splicing primers were run on an Agilent Bioanalyzer for quantitative analysis. Primer pairs used are listed below in Table 2.

### 4.5. Conditional Mouse Design and Genotyping

The mouse *qkI* conditional null allele was reported previously [3]. The final genomic organization is depicted in Figure 3A with two loxP sequences flanking exon 2 of the *qkI* gene. Ear biopsies were used to isolate gDNA using the AccuStart II PCR Genotyping Kit (Quanta-Bio, Beverly, MA, USA) for genotyping using primers listed in Table 3.

### 4.6. Immunohistochemistry

Fluorescence immunostainings were performed on mouse kidney cryosections that were obtained by freezing tissue samples embedded in O.C.T. (TissueTek^®^, Torrance, CA, USA) and placing the mold in an isopentane solution cooled with liquid nitrogen. Next, 4 μm sections were cut using a cryostat (Leica, Wetzler, Germany), slides were air-dried for 30 min, then fixed in acetone for 10 min at room temperature, air-dried again for 30 min and stored at −20 °C. Before staining, slides were washed three times in PBS at room temperature and incubated in blocking buffer (PBS + 1%BSA + 1% FCS) for at least 1 h at room temperature. Slides were incubated with primary antibodies in blocking buffer for at least 3 h at room temperature or at 4 °C overnight, where after extensive washing in PBS followed and subsequently incubated with the appropriate Alexa^®^ (Invitrogen) labeled secondary antibodies in blocking buffer. Embedding the slides was performed in Prolong gold (Invitrogen) containing DAPI. Imaging was performed on a Leica DM5500 or a Leica SP5 confocal imaging system.

Histochemistry analysis for collagen content was done using Picosirius red staining on formalin-fixed, paraffin-embedded tissues. CLK and UUO kidneys were fixed in 3.7% formalin in PBS for 2 h then put on 70% ethanol and subsequently dehydrated overnight followed by paraffin embedding according to standard protocols. For analyses, 4 μm sections were prepared. Prior to staining, slides were brought to water by first “de-paraffinizing” in Xylene, then taken through a series of solutions decreasing in ethanol percentage (100% to 50%) in 10% steps taking 5 min at a time, finally incubation in H_2_O for 30 min. Slides were submerged in Sirius red F3B solution (0.1% Direct Red 80, Sigma Aldrich) in a saturated aqueous solution of picric acid for 1 h at room temperature. Slides were washed in 3 stages of acidified water consisting of 5 mL glacial acetic acid (Millipore-Sigma, Carlsbad, CA, USA) in 1 L of water. After this, slides were dehydrated to 100% ethanol and thereafter Xylene, then embedded in Entellan. Images were taken using a Leica DMI4400B microscope and collagen deposition was quantified using ImageJ [62]. Outliers were removed using a statistical outlier test.

### 4.7. Statistical Analysis

All data were statistically tested using a two-tailed Students’ *t*-test. Error bars in all figures represent SEM.

### 4.8. Mouse Studies

All mouse studies were approved and performed according to the guidelines of the relevant authorities of the Leiden University Medical Center, Leiden University in The Netherlands or the Lady Davis Institute, McGill University, Montreal, Canada. Unilateral Urethral Obstruction was performed as described earlier [27].

## Figures and Tables

**Table 1:** Antibodies.

Antibody	Manufacturer	Cat. Nr.	Application
Rabbit α-QKI-5	Millipore	AB9904	WB + IF
Rabbit α-QKI-6	Millipore	AB9906	IF
Rabbit α-QKI-7	Millipore	AB9908	IF
Rat α-Meca-32	BD Pharmingen	550563	IF
Rat α-F4/80	Abcam	ab6640	IF
Mouse α-Pan-QKI	Neuromab	75–168	WB
Mouse α-QKI-5	Neuromab	73–232	WB
Mouse α-QKI-6	Neuromab	73–190	WB
Mouse α-QKI-7	Neuromab	73–200	WB
Rabbit α-CD206	Abcam	ab64693	WB
Mouse α-αSMA	Abcam	ab7817	WB
Rabbit α-HistoneH3	Abcam	ab1791	WB

**Table 2:** Primers for reverse transcription PCR.

mRNA Expression and Splicing Primers	5′–3′
FW-QKI5	GTGTATTAGGTGCGGTGGCT
REV-QKI5	ATAGGTTAGTTGCCGGTGGC
FW-QKI6	ACCTAGTGGTGTATTAGGTATGGCT
REV-QKI6	CCGGAGGCTGCTGAGACTA
FW-QKI7	ACATTGGCACCAGCTACATCA
REV-QKI7	CAGCAAGTCAATGGGCTGAAAT
FW-EMR1	CCTGGACGAATCCTGTGAAG
REV-EMR1	GGTGGGACCACAGAGAGTTG
FW-CD115	AGAGTGATGTGTGGTCCTAC
REV-CD115	GTTAGCATAGTCCTGGTCTC
FW-COL1A1	TGACTGGAAGAGCGGAGAGT
REV-COL1A1	GTTCGGGCTGATGTACCAGT
FW-aSMA	CTGACAGAGGCACCACTGAA
REV-aSMA	CATCTCCAGAGTCCAGCACA
FW-exon2-QKI	GGTGGGACCCATTGTTCAGT
REV-exon5-QKI	AGGCTGTCTTCACCTTCAGC
FW-GAPDH	ACTCCCACTCTTCCACCTTC
REV-GAPDH	CACCACCCTGTTGCTGTAG
FW-ACTB	AGGTCATCACTATTGGCAACGA
REV-ACTB	CCAAGAAGGAAGGCTGGAAAA
FW-ADD3-splicing	CCACCTCCTGGAAGGAGAAC
REV-ADD3-splicing	CATGGAGGTGAAGCTCTTGGA
FW-PTPRO-splicing	ATGTGGAGCTGGCACGTTTG
REV-PTPRO-splicing	ACGGGGTTTGTTAGTTTCCTCT
FW-FGFR1OP2-splicing	CATGGCCAGCAAGAAAGATGAC
REV-FGFR1OP2-splicing	TTTGGTCAACATGTGCTTGC
FW-REPS1-splicing	AGCCAGGTGAGGTAGGTTACT
REV-REPS1-splicing	CTGCATGTGGATTTTGCTTGGA

**Table 3:** QKI genotyping primers.

Genotyping Primers	5′–3′
FW-QKI-flox	ACAGAGGCTTTTCCTGACCA
REV-QKI-flox	TTCAGAACCCCCACATTACC
FW-QKI-recombination	CCTGGAATGGTGCTTTCCTA
REV-QKI-recombination	TTCAGAACCCCCACATTACC
FW-quaking viable genotyping	TCTAAAGAGCATTTTCGAAGT
REV-quaking viable genotyping	TTGCTAACTGAATATTACT
